# Association of HPA axis-related genetic variation with stress reactivity and aggressive behaviour in pigs

**DOI:** 10.1186/1471-2156-11-74

**Published:** 2010-08-09

**Authors:** Eduard Muráni, Siriluck Ponsuksili, Richard B D'Eath, Simon P Turner, Esra Kurt, Gary Evans, Ludger Thölking, Ronald Klont, Aline Foury, Pierre Mormède, Klaus Wimmers

**Affiliations:** 1 Research Unit "Molecular Biology", Leibniz Institute for Farm Animal Biology (FBN), Wilhelm-Stahl-Allee 2, D-18196 Dummerstorf, Germany; 2 Research Group "Functional Genomic", Leibniz Institute for Farm Animal Biology (FBN), Wilhelm-Stahl-Allee 2, D-18196 Dummerstorf, Germany; 3 Sustainable Livestock Systems, SAC, West Mains Road, Edinburgh, EH9 3JG, UK; 4 Optimeater Consulting, Straatsburglaan 18, Mol, Belgium; 5 PIC UK, 2 Kingston Business Park, Kingston Bagpuize, Oxfordshire, OX13 5FE, UK; 6 PIC Germany, PIC Deutschland GmbH, Ratsteich 31, 24837 Schleswig, Germany; 7 Université Victor Segalen Bordeaux 2, PsyNuGen, UMR1286 INRA, 33076 Bordeaux, France

## Abstract

**Background:**

Stress, elicited for example by aggressive interactions, has negative effects on various biological functions including immune defence, reproduction, growth, and, in livestock, on product quality. Stress response and aggressiveness are mutually interrelated and show large interindividual variation, partly attributable to genetic factors. In the pig little is known about the molecular-genetic background of the variation in stress responsiveness and aggressiveness. To identify candidate genes we analyzed association of DNA markers in each of ten genes (*CRH *g.233C>T, *CRHR1 *c.*866_867insA, *CRHBP *c.51G>A, *POMC *c.293_298del, *MC2R *c.306T>G, *NR3C1 *c.*2122A>G, *AVP *c.207A>G, *AVPR1B *c.1084A>G, *UCN *g.1329T>C, *CRHR2 *c.*13T>C) related to the hypothalamic-pituitary-adrenocortical (HPA) axis, one of the main stress-response systems, with various stress- and aggression-related parameters at slaughter. These parameters were: physiological measures of the stress response (plasma concentrations of cortisol, creatine kinase, glucose, and lactate), adrenal weight (which is a parameter reflecting activity of the central branch of the HPA axis over time) and aggressive behaviour (measured by means of lesion scoring) in the context of psychosocial stress of mixing individuals with different aggressive temperament.

**Results:**

The SNP *NR3C1 *c.*2122A>G showed association with cortisol concentration (p = 0.024), adrenal weight (p = 0.003) and aggressive behaviour (front lesion score, p = 0.012; total lesion score p = 0.045). The SNP *AVPR1B *c.1084A>G showed a highly significant association with aggressive behaviour (middle lesion score, p = 0.007; total lesion score p = 0.003). The SNP *UCN *g.1329T>C showed association with adrenal weight (p = 0.019) and aggressive behaviour (front lesion score, p = 0.029). The SNP *CRH *g.233C>T showed a significant association with glucose concentration (p = 0.002), and the polymorphisms *POMC *c.293_298del and *MC2R *c.306T>G with adrenal weight (p = 0.027 and p < 0.0001 respectively).

**Conclusions:**

The multiple and consistent associations shown by SNP in *NR3C1 *and *AVPR1B *provide convincing evidence for genuine effects of their DNA sequence variation on stress responsiveness and aggressive behaviour. Identification of the causal functional molecular polymorphisms would not only provide markers useful for pig breeding but also insight into the molecular bases of the stress response and aggressive behaviour in general.

## Background

Stress responses promote the maintenance of homeostasis and adaptation to physiological and psychosocial challenges of a changing environment. This complex process involves coordinated activation of behavioural, autonomic, and neuroendocrine reactions. Concomitantly, pathways that promote vegetative functions such as growth, reproduction, and feeding are inhibited, the extent to which is dependent upon the duration and intensity of the stressor ("biological cost" of the stress response [1]).

Aggression is a powerful stressor and has been shown to activate the hypothalamic-pituitary-adrenocortical (HPA) axis as well as the sympatho-adrenomedullar (SAM) system in various species including pigs [2-4]. In the pig, aggression commonly occurs when mixing unfamiliar individuals which disturbs the social dominance order [5]. Besides negative effects on animal welfare, aggression has also been shown to have a negative impact on immune response [6], growth performance [7], and product quality in pigs [4,8]. Aggressive behaviour in turn is affected by the functional properties of the HPA axis and of the SAM system (reviewed in [9]). Baseline levels of glucocorticoids are inversely related with aggressiveness in various species including pigs (reviewed in [9,6,10]). In contrast, as reported in rodents, an acute increase in glucocorticoid levels may promote aggressive behaviour by a fast feed forward mechanism [11].

Functional properties of the stress response systems and aggressive behaviour show large interindividual variation, depending upon a variety of factors including genetic predisposition [12-14]. In humans and in model animals several genomic regions and gene variants associated with variation in stress responsiveness and aggressiveness have been identified using quantitative trait loci (QTL) mapping and candidate gene approaches (reviewed in [15-18]). In the pig, such studies are scarce, in spite of the widely recognized impact of aggressive behaviour and stress on the expression of meat and carcass quality traits [8]. Désautés *et al. *[19] mapped QTL for behavioural and neuroendocrine stress responses in a Meishan × Large White intercross leading to the identification of the corticosteroid binding globulin encoding gene as a major QTL for plasma cortisol levels in this population ([20], reviewed in [18]). In addition Geldermann *et al. *[21] mapped QTL for plasma creatine kinase levels after pharmacological challenge. Using the candidate gene approach Fujii *et al. *[22] identified a mutation (SNP c.1843C>T) in the ryanodine receptor 1 (*RYR1*) gene responsible for malignant hyperthermia and porcine stress syndrome. The mutation has been shown to affect the basal functioning of the HPA axis *in vivo *[23] and *in vitro *[24], and analysis of this important mutation was included in the present study.

The aim of the present study was to expand the current knowledge of the molecular-genetic basis of the variation in stress responsiveness and aggressive behaviour in the pig. To this end we analyzed the association of candidate gene DNA markers with physiological parameters of the stress response (cortisol, creatine kinase, glucose, and lactate concentration in plasma) and aggressive behaviour (measured by means of lesion scoring) in the context of the psychosocial stress of mixing individuals with different aggressive temperaments in a commercial pig herd [4]. In addition, we also analyzed the association with adrenal weight, a parameter reflecting activity of the central branch of the HPA axis (i.e. the release of corticotropin-releasing hormone and ACTH) over time, in a different herd of commercial crossbred pigs. To relate position of the candidate genes with known porcine QTL we physically mapped those candidates whose position had not previously been determined.

The candidates represented genes encoding members of the HPA axis pathway (corticotropin-releasing hormone, *CRH; *CRH type 1 receptor, *CRHR1*; CRH binding protein, *CRHBP*; vasopressin, *AVP*; vasopressin V_1B _receptor, *AVPR1B*; proopiomelanocortin, *POMC*; melanocortin type 2 (ACTH) receptor, *MC2R*; glucocorticoid receptor, *NR3C1*) and genes encoding members of the corticotropin-releasing hormone (CRH) system (urocortin, *UCN; *CRH type 2 receptor, *CRHR2*). CRH and vasopressin act synergistically to activate expression of proopiomelanocortin, the precursor of adrenocorticotropin (ACTH), in the pituitary through activation of CRH type 1 and vasopressin V_1B _receptors respectively. CRH is the dominant trigger for HPA axis activation during acute stress while vasopressin, which itself is a weak ACTH secretagogue, is important in mediating response to chronic stress. Vasopressin also has an important function in the control of aggressive behaviour in various species [25] including the pig [26]. CRH binding protein functions as a buffer for CRH and related peptides and plays an inhibitory role in the modulation of CRH activity. ACTH stimulates synthesis and secretion of glucocorticoids from the adrenal cortex via melanocortin type 2 receptor. The action of glucocorticoids on target tissues is mediated by glucocorticoid receptor, which in the hypothalamus, in the pituitary, and in the hippocampus acts to terminate the stress response (reviewed in [27]). The urocortins, although not directly involved in the HPA axis action, may play a modulatory role via stimulation of the CRH receptor type 2 [28].

## Results and Discussion

### Allele distribution of the candidate genes

The candidate gene DNA markers are located mainly in the transcribed region (Table 1) with the exception of the SNPs *CRH *g.233C>T and *UCN *g.1329T>C, where the DNA markers are located in the promoter region in evolutionarily conserved segments, ~350bp and ~90bp upstream of the transcription start site respectively ([29] and unpublished data respectively). Two DNA markers lead to amino acid exchanges (*CRHBP *c.51G>A, *AVPR1B *c.1084A>G), and one to an amino acid deletion (*POMC *c.293_298del), respectively. The remaining markers are either silent SNPs (*MC2R *c.306T>G, *AVP *c.207A>G) or polymorphisms located in the 3' untranslated region (*CRHR1 *c.*866_867insA, *CRHR2 *c.*13T>C, *NR3C1 *c.*2122A>G). The SNP c.306T>G in *MC2R *was reported previously by others [30].

**Table 1:** *RYR1 *and candidate gene DNA marker frequencies in different lines

Gene	**Position**^2,3^	Allele	**SYN**^4^	PiF1a	LR	LW	Pi	WB	MK
**SNP designation**^1^			n = 427	n = 208	n = 24	n = 26	n = 25	n = 22	n = 17
***RYR1 *c.1843C>T**	coding (p.R615C)^a^	C	*0.83*	*0.75*	NA	NA	0.58	NA	NA
***CRH*** **g.233C>T**	promoter^b^	C	0.59	0.54	0.67	0.52	0.47	0.48	1.00
***CRHR1*** c.*866_867insA	3'UTR^c^	Del	0.54	0.42	0.71	0.58	0.50	1.00	0.32
***CRHBP*** **c.51G>A**	coding (p.M17I)^c^	G	0.92	0.92	0.92	0.77	0.92	1.00	0.84
***POMC *c.293_298del**	coding (p.G96_G97del)^c^	Ins	0.98	0.95	0.94	0.96	1.00	1.00	0.03
***MC2R*** **c.306T>G**	coding (silent)^d^	T	0.11	0.17	0.44	0.02	0.16	0.00	0.03
***NR3C1*** c.*2122A>G	3'UTR^c^	A	0.49	0.50	0.48	0.21	0.42	0.05	1.00
***AVP*** **c.207A>G**	coding (silent)^c^	A	0.66	0.62	0.65	0.58	0.80	0.66	0.97
***AVPR1B*** **c.1084A>G**	coding (p.S362G)^c^	A	*0.89*	0.83	0.65	0.90	0.90	1.00	0.24
***UCN*** **g.1329T>C**	promoter^c^	T	*0.88*	0.84	0.94	0.73	0.94	1.00	0.59
***CRHR2*** c.*13T>C	3' UTR^c^	T	0.49	0.59	0.54	0.73	0.38	0.95	0.81

^1^SNP designation according to the HGVS nomenclature. Reference sequence is given in Additional File 1, Table S1-S4

^2^Gene region where the analyzed polymorphism is located. For polymorphisms in the coding region the effect on protein sequence is given in parenthesis.

^3^Reference: ^a^[22], ^b^[29], ^c^dbSNP submitter accession numbers listed in Additional File 1, Table S1-S4, ^d^[30]

^4^Lines: SYN-synthetic, PiF1a-Pietrain × (German Large White × German Landrace), LR-German Landrace, LW-German Large White, Pi-Pietrain, WB-Wild Boar, MK-Muong Khuong

Underline: the genotype distribution departures from Hardy-Weinberg equilibrium at p < 0.05

Italic underline: the genotype distribution departures from Hardy-Weinberg equilibrium at p < 0.01

NA-not analyzed

Allele frequencies of the candidate gene DNA markers in the two phenotyped commercial crossbred herds (SYN and PiF1a), along with allele frequencies in sets of pigs from three commercial pure breeds German Landrace (LR), German Large White (LW), and Pietrain (Pi), in a set from Vietnamese local breed Muong Khuong (MK), and in a set of European Wild Boars (WB) are summarized in Table 1. Contrary to the common assumption that selective breeding reduces genetic variability, the commercial breeds and crosses showed the highest variability, whereas Wild Boar the least. Six out of the ten candidate gene DNA markers tested, namely *CRHR1 *c.*866_867insA, *CRHBP *c.51G>A, *AVPR1B *c.1084A>G, *POMC *c.293_298del, *MC2R *c.306T>G, and *UCN *g.1329T>C were fixed in Wild Boar. The increased variability of the commercial breeds compared to Wild Boar may be a result of the introgression of Asian genetics into European breeds in 18^th ^and 19^th ^centuries. Ramirez *et al. *[31] showed that the proportion of Asian genetics in the genetic pool of European commercial breeds is still significant at around 47-61%. The majority of the candidate gene DNA markers, as for example the SNP *AVPR1B *c.1084A>G, showed large differences in allele distribution between Wild Boar and Muong Khuong, indicating that the alleles missing in Wild Boar but segregating in commercial breeds may originate from the introgression of the Asian genetics.

In view of the intriguing differences in allele frequency of the DNA markers of the HPA axis-related genes between commercial, local, and wild pigs it is interesting to note that domestication of several species and selection for tameness (i.e. reduced interspecific aggression) in silver foxes and rats, which is an important aspect of domestication, was coupled with decreased activity of the HPA axis ([32], reviewed in [33]). Likewise, domestic pig breeds show lower cortisol levels compared to their wild ancestor [34]. Although the reduced cortisol level in modern pigs may result from selection for leaner body composition [35], it is conceivable that it might be related to domestication-related behaviour as well.

A test of Hardy-Weinberg equilibrium revealed significant deviation in both crossbred herds for the SNPs *RYR1 *c.1843C>T and *AVPR1B *c.1084A>G, for the SNPs *AVP *c.207A>G and *UCN *g.1329T>C in SYN, and for the SNP *NR3C1 *c.*2122A>G in PiF1a. In contrast in the pure breeds, only the polymorphisms *CRHR1 *c.*866_867insA in Pietrain and *CRHR2 *c.*13T>C in Muong Khuong showed significant deviation, i.e. in two tests out of fifty performed, which is a proportion that would be expected to occur at the significance level of 5% simply by chance. The more frequent deviation from Hardy-Weinberg equilibrium in SYN and PiF1a is thus likely a result of crossbreeding, rather than an indication of selection.

### Association with stress responsiveness and aggressive behaviour

The physiological parameters of the stress response included in this study were shown previously to be affected by aggressive interactions [2,36] and also in the present experiment as reported earlier [4]. In this context creatine kinase (CK), lactate and glucose concentration reflect the amount of physical activity and mobilization of energy sources, and indicate activation of the SAM system, whilst cortisol level indicates activation of the HPA axis. For evaluating aggressive behaviour at mixing we used the number of skin lesions, which is an established indicator trait. Turner *et al. *[14,37,38] showed that front lesion score is the best indicator of engagement in reciprocal aggression at mixing, whereas rear lesion score is the best indicator of receipt of nonreciprocal aggression at mixing.

Effects with p ≤ 0.10 are summarized in Tables 2 and 3 for stress parameters and in Table 4 for lesion scores. We refer to results as significant when p < 0.05 and as showing a tendency when 0.05 ≤ p ≤ 0.10. To advise the reader of the increased risk of type I error due to multiple testing we also provide the corresponding false discovery rate (q-value; Tables 2, 3, 4). The q value is similar to the well known p value, except it is a measure of significance in terms of the false discovery rate rather than the false positive rate [39].

**Table 2:** Association of candidate gene DNA markers with physiological stress parameters in the synthetic (SYN) line

Gene	Genotype		Genotype		Genotype		
Trait*	**LSM**^1,2^	**SE**^1^	LSM	SE	LSM	SE	p-value
	(n)		(n)		(n)		**q-value**^3^
***RYR1 *c.1843C>T**	**CC**		**CT**		**TT**		
**Glucose (mmol/l)**	13.9	0.33	13.0	0.44	na^4^		0.038
	(275)		(140)				0.21
**Lactate (mmol/l)**	10.1	0.23	11.5	0.31	na		<0.0001
	(276)		(140)				<0.0001
**CK (log_10_(U/l))**	3.55	0.02	3.79	0.03	na		<0.0001
	(275)		(135)				<0.0001
**Cortisol (ng/ml)**	53.7	1.40	49.2	1.89	na		0.020
	(276)		(140)				0.17
							
***CRH *g.233C>T**	**CC**		**CT**		**TT**		
**Glucose (mmol/l)**	13.8^e^	0.40	12.8^a,f^	0.39	14.6^b^	0.58	0.002
	(143)		(204)		(65)		0.04
**CK (log_10_(U/l))**	3.63^e^	0.03	3.69	0.03	3.73^f^	0.04	0.055
	(142)		(199)		(66)		0.27
							
*NR3C1 *c.*2122A>G	**AA**		**AG**		**GG**		
**Cortisol (ng/ml)**	46.6^c^	2.27	52.1^d^	1.58	52.7^d^	2.02	0.024
	(92)		(210)		(96)		0.18

^1^LSM: least square mean; SE: standard error

^2 ^LSM with different superscripts ^a,b; c,d; e,f ^differ at p < 0.01; p < 0.05; and p < 0.1 respectively

^3^q-value: false discovery rate

^4^na: not available or not analyzed due to low frequency n < 10;

**Table 3:** Association of candidate gene DNA markers with weight of the adrenal gland in the PiF1a line

Gene	Genotype		Genotype		Genotype		
Trait*	**LSM**^1,2^	**SE**^2^	LSM	SE	LSM	SE	p-value
	(n)		(n)		(n)		**q-value**^3^
***RYR1 *c.1843C>T**	**CC**		**CT**		**TT**		
**Adrenal Weight (g)**	2.29	0.03	2.22	0.03	na^4^		0.073
	(98)		(107)				0.31
							
***POMC *c.293_298del**	**InsIns**		**InsDel**		**DelDel**		
**Adrenal Weight (g)**	2.24	0.02	2.40	0.07	na		0.027
	(179)		(22)				0.19
							
***MC2R *c.306T>G**	**TT**		**TG**		**GG**		
**Adrenal Weight (g)**	2.11	0.04	2.31	0.03	na		<0.0001
	(59)		(142)				<0.0001
							
*NR3C1 *c.*2122A>G	**AA**		**AG**		**GG**		
**Adrenal Weight (g)**	2.11^a^	0.05	2.29^b^	0.03	2.30^b^	0.05	0.003
	(42)		(119)		(45)		0.04
							
***UCN *g.1329T>C**	**TT**		**TC**		**CC**		
**Adrenal Weight (g)**	2.21	0.03	2.33	0.04	na		0.019
	(146)		(54)				0.17
							
*CRHR2 *c.*13T>C	**TT**		**TC**		**CC**		
**Adrenal Weight (g)**	2.31^e^	0.04	2.24	0.03	2.18^f^	0.05	0.087
	(77)		(91)		(38)		0.33

^1^LSM: least square mean; SE: standard error

^2^LSM with different superscripts ^a,b; c,d; e,f ^differ at p < 0.01; p < 0.05; and p < 0.1 respectively

^3^q-value: false discovery rate

^4^na: not available or not analyzed due to low frequency n < 10;

**Table 4:** Association of candidate gene DNA markers with aggressive behaviour in the synthetic (SYN) line

Gene	Genotype		Genotype		Genotype		p-value
Trait	**LSM**^1,2^	**SE**^1^	LSM	SE	LSM	SE	**q-value**^3^
	(n)		(n)		(n)		
***RYR1 *c.1843C>T**	**CC**		**CT**		**TT**		
**Rear Lesions (log_10 _LS)**	0.87	0.027	0.95	0.035	na^4^		0.032
	(255)		(132)				0.19
							
***CRH *g.233C>T**	**CC**		**CT**		**TT**		
**Rear Lesions (log_10 _LS)**	0.86^e^	0.035	0.92	0.031	0.99^f^	0.051	0.085
	(134)		(189)		(61)		0.33
							
***CRHBP *c.51G>A**	**GG**		**GA**		**AA**		
**Middle Lesions (log_10 _LS)**	1.22	0.026	1.31	0.051	na		0.060
	(329)		(69)				0.27
							
*NR3C1 *c.*2122A>G	**AA**		**AG**		**GG**		
**Total Lesions (log_10 _LS)**	1.69^e^	0.035	1.68^e^	0.024	1.59^f^	0.033	0.045
	(80)		(174)		(83)		0.24
**Front Lesions (log_10 _LS)**	1.15^c^	0.046	1.12^c^	0.032	0.99^d^	0.044	0.012
	(85)		(190)		(88)		0.13
							
***AVPR1B *c.1084A>G**	**AA**		**AG**		**GG**		
**Total Lesions (log_10 _LS)**	1.68	0.021	1.57	0.034	na		0.003
	(271)		(82)				0.04
**Front Lesions (log_10 _LS)**	1.11	0.028	1.02	0.047	na		0.061
	(295)		(86)				0.27
**Middle Lesions (log_10 _LS)**	1.25	0.026	1.13	0.045	na		0.007
	(309)		(90)				0.08
							
***UCN *g.1329T>C**	**TT**		**TC**		**CC**		
**Front Lesions (log_10 _LS)**	1.07	0.028	1.17	0.045	na		0.029
	(285)		(90)				0.19
							

^1^LSM: least square mean; SE: standard error

^2^LSM with different superscripts ^a,b; c,d; e,f ^differ at p < 0.01; p < 0.05; and p < 0.1 respectively

^3^q-value: false discovery rate

^4^na: not available or not analyzed due to low frequency n < 10;

All physiological stress parameters were significantly affected by the *RYR1 *SNP c.1843C>T (Table 2). In accordance with the known effect of the *RYR1 *SNP on metabolism of the skeletal muscle [40,41] the mutated T allele highly significantly (p < 0.0001) increased plasma CK and lactate concentration. Plasma glucose concentration, in turn, was reduced. Consistent with Weaver *et al. *[23], heterozygous carriers of the T allele showed significantly lower cortisol concentration compared to CC homozygous individuals. Furthermore the adrenal weight tended to be lower in the heterozygous carriers of the T allele in the PiF1a line, providing independent supporting evidence that the *RYR1 *SNP affects activity of the HPA axis (Table 3). With increased sample size this effect became significant (n = 316; data not shown). Concerning aggressive behaviour the T allele of the *RYR1 *SNP c.1843C>T significantly increased rear lesion score (Table 4). The effect of *RYR1 *on aggressive behaviour might be related to its effect on HPA axis activity. Guárdia *et al. *[42] also reported an effect of *RYR1 *on skin lesions, but the direction of the effects was opposite to our findings, perhaps due to differences in recording of the skin lesions (in the study of Guárdia *et al. *[42] as a whole carcass score on a 5-point scale).

The SNP *CRH *g.233C>T showed a significant association with plasma glucose concentration and a tendency to affect plasma CK concentration; however the effect on glucose showed neither an additive nor dominance direction (Table 2). We mapped *CRH *previously on SSC4 in the marker interval SW724-S0107 [29]. So far no QTL for physiological stress parameters have been reported in this genomic region. The effect on CK concentration is possibly related to aggressive behaviour, because carriers of the T allele, which tended to have higher CK concentration, also tended to have a higher rear lesion score (Table 4). It could be speculated that the association of the SNP *CRH *g.233C>T with aggressive behaviour might be related to the anxiogenic effect of CRH [43,44] because the SNP showed no association with activity of the HPA axis. The anxiogenic effect of CRH is, at least partly, independent of its action on the HPA axis [45]. Anxiety is related to aggressive behaviour in a complex manner, depending on the model used. Veenema and Neumann [46] for example, found an inverse relationship between anxiety and offensive aggression in rats divergently selected for anxiety-related behaviour.

*CRHR1 *maps on SSC12 in the marker interval SW957-SW943 (Additional File 1, Table S6). No effects, on either the stress parameters or on aggressive behaviour were detected for the polymorphism *CRHR1 *c.*866_867insA. *CRHBP *maps on SSC2 in the marker interval SW1602-SW1320 (Additional File 1, Table S6). The SNP *CRHBP *c.51G>A showed a tendency to affect middle lesion score (Table 4), but showed no association with physiological stress parameters or adrenal weight.

The polymorphisms *POMC *c.293_298del and *MC2R *c.306T>G both showed association only with adrenal weight in the PiF1a line (Table 3). This effect is in line with the established positive effect of *POMC*-derived peptides, in particular ACTH, on adrenal growth [47]. We mapped *POMC *on SSC3, in the marker interval SW314-S0002 (Additional File 1, Table S6), close to a QTL for basal glucose level [19]. *POMC *is involved in the control of glucose homeostasis via the HPA axis and other pathways [48], however in the present study we found no association of the polymorphism *POMC *c.293_298del with glucose concentration. *MC2R *was mapped on SSC6 close to marker SW2173 by Jacobs *et al. *[30] and close to QTL for plasma creatine kinase levels in the Meishan × Pietrain and Wild Boar × Pietrain families [49]. However these QTL are most likely caused by the SNP c.1843C>T in *RYR1*, which is also located in the same QTL region.

The SNP *NR3C1 *c.*2122A>G showed a significant association with plasma cortisol concentration in SYN and a significant association with adrenal weight in PiF1a (Tables 2 and 3). The allele A, which is associated with lower cortisol concentration, is also associated with lower adrenal weight (Tables 2 and 3), suggesting that it is associated with an enhanced negative feedback effect on the activity of the HPA axis. A similar phenotype, including decreased basal plasma corticosterone and ACTH levels, reduced adrenal weight and adrenocortical size, has been reported in a knock-in mouse line showing increased functioning of a modified glucocorticoid receptor [50]. A clue about possible molecular mechanisms underlying the enhanced negative feedback effect might be obtained from the report of Perreau *et al. *[51] who observed a higher density of glucocorticoid receptors in the pituitary in Large White pigs which exhibit lower activity of the HPA axis compared to Meishan pigs, suggesting that genetic factors cause variation in glucocorticoid receptor density in the pituitary of the pig. Furthermore, the SNP *NR3C1 *c.*2122A>G showed a significant association with the total and front lesion score, but not with the middle or rear lesion score (Table 4). This indicates that the animals accumulated lesions mainly through reciprocal fighting. Because, as mentioned in the introduction, baseline levels of glucocorticoids are inversely related with aggressiveness, the decreased activity of the HPA axis in the carriers of the allele A provides a possible explanation for their enhanced aggressive behaviour. *NR3C1 *maps on SSC2 in the marker interval SW1879-SWR308 (Additional File 1, Table S6). So far no QTL for physiological stress parameters have been reported in this genomic region.

*AVP *maps on SSC17 in a QTL region for post-stress ACTH level [19]. However, in the present study the SNP *AVP *c.207A>G showed associations with neither the physiological stress parameters, nor with aggressive behaviour. The SNP *AVPR1B *c.1084A>G in turn showed significant association only with aggressive behaviour (Table 4). The allele G consistently decreased lesion score. However, the effect was most pronounced for the middle lesion score, less pronounced for the front lesion score, and did not reach the 0.1 level for the rear lesion score (Table 4). The vasopressin pathway plays a prominent role in the regulation of social behaviour, including aggression [25,26]. Vasopressin V_1B _receptor knockout mice display reduced levels of social forms of aggressive behaviour. While male vasopressin V_1B _receptor knockout mice demonstrate deficits in offensive and defensive aggression, female knockout mice have deficits in maternal aggression. However, there is no global deficit in aggressive behaviour as vasopressin V_1B _receptor knockout mice show normal predatory aggression [52]. Hence, there is strong functional evidence supporting the identified association of the SNP *AVPR1B *c.1084A>G with aggressive behaviour in the context of social stress of mixing. *AVPR1B *maps on SSC9 in the marker interval SW1879-SWR308 (Additional File 1, Table S6).

The SNP *UCN *g.1329T>C showed no association with physiological stress parameters but a significant association with adrenal weight in PiF1a (Table 3) indicating that it might affect activity of the HPA axis. Urocortin is thought to be involved in the autonomic stress response [53] but so far studies with knockout mouse models have revealed no consistent evidence for an involvement of urocortin in the HPA axis response to acute stress (reviewed in [54]). The study of Zalutskaya *et al. *[55] on the response of urocortin knockout mice to repeated restraint indicates that urocortin may play a role in the adaptation of the HPA axis to chronic stress. Little is known about the function of urocortin in the pig. Parrott *et al. *[43] showed that intracerebroventricular injection of urocortin increases cortisol release in the pig. Besides association with adrenal weight the SNP *UCN *g.1329T>C also showed significant association with front lesion score (Table 4). Urocortin, similar to CRH, possess an anxiogenic effect [43,56]. As discussed above for the SNP *CRH *g.233C>T, this might also underlie the association of the SNP *UCN *g.1329T>C with aggressive behaviour. *UCN *maps on SSC3 in the marker interval SW730-SWR201 (Additional File 1, Table S6), close to the position of *POMC *and to a QTL for basal glucose level [19]. Urocortins are involved in the regulation of glucose homeostasis [57,58]. However in the present study we found no association of the SNP *UCN *g.1329T>C with the glucose concentration.

*CRHR2 *maps on SSC18 in the marker interval SW787-SW1682 (Additional File 1, Table S6). The only effect we found for the SNP *CRHR2 *c.*13T>C was a tendency for adrenal weight in PiF1a (Table 3). On SSC18 QTL for basal and stress-induced increase in cortisol level were detected [19], however these map distal to *CRHR2*.

In the present study to examine phenotypic effects of the HPA axis-related genes we used a single DNA marker per gene. However, the HPA axis-related genes are usually highly polymorphic with several polymorphisms affecting gene expression and/or function of the encoded protein [29,59,60], with the consequence that a single DNA marker most likely does not capture all of the genetic information. Furthermore, for several polymorphisms, e.g. for *POMC *c.293_298del or *CRHBP *c.51G>A, the power to detect an association was limited by the low frequency of the minor allele. Therefore, genes that showed no significant associations here could still harbour functional DNA sequence variation with phenotypic effects. On the other hand the SNP in *NR3C1 *and *AVPR1B *showed multiple consistent effects, partly significant even after correction for multiple testing (the SNP *NR3C1 *c.*2122A>G on adrenal weight and the SNP *AVPR1B *c.1084A>G on middle lesion score respectively), providing convincing evidence for a genuine effect of the DNA sequence variation of these two genes on stress responsiveness and aggressive behaviour. Consequently, the SNP used here are either directly involved or are in linkage disequilibrium with the causal variants.

## Conclusions

In the pig knowledge about the molecular basis of the stress response, aggressive behaviour and their interindividual variation is very limited. In the present study we analyzed the association between DNA markers of ten HPA axis related genes with stress reactivity and aggressive behaviour in the context of psychosocial stress of mixing individuals with different aggressive temperaments. From this we obtained convincing evidence for an effect of two genes: *NR3C1 *on HPA axis activity and aggressive behaviour, and *AVPR1B *on aggressive behaviour. Our results provide a foundation for future studies directed at the identification of the causal functional DNA sequence variation which would not only provide markers useful for pig breeding but also insight into the molecular basis of the stress response and aggressive behaviour in general.

## Methods

### Animals

The structure and phenotyping of the pig line designated SYN (synthetic) was described in detail by D'Eath *et al. *[4]. Briefly, pigs of the SYN line were progeny of Pietrain sires and crossbred dams from six different commercial parent lines of the Pig Improvement Company (PIC). The *RYR1 *SNP c.1843C>T was segregating among the Pietrain sires. Aggressive temperament was measured by counting skin lesions (lesion scoring) immediately before and 24 h after mixing pigs into new groups at approximately 10-11 weeks of age. The increase in number of skin lesions has been shown by Turner *et al. *[14] to be positively associated with the duration of involvement in reciprocal fighting behaviour. Pigs in each mixed group were ordered by the change in total skin lesions: half of the pigs in each group (those with the most lesions) were designated as high aggressiveness and the remaining half as low aggressiveness. In four slaughter batches pigs were assigned to one of four mixing treatments based on their aggressiveness (number of animals per treatment included in this study: high with high n = 63, high with low n = 61, low with low n = 65, and unmixed n = 64). Pigs were mixed into their treatment groups as they were loaded onto a vehicle for transport to the abattoir. In a fifth treatment, experienced by another four batches, mixing of pigs occurred at loading onto the truck and at lairage in an uncontrolled way, typical of commercial practice (n = 165). Skin lesions were counted before mixing and after slaughter on the carcass, dividing the body into front (head, neck, shoulders and front legs), middle (flanks and back) and rear (rump, hind legs and tail) sections. The difference in lesion number was again taken as the lesion score.

The mixing treatment had significant effect on aggressive behaviour as reported by D'Eath *et al. *[4]. The animal experiment received approval from the Scottish Agricultural College Animal Experiments Committee.

Pigs were stunned by means of CO_2 _gas and at exsanguination, a 50 ml sample of trunk blood was collected from each pig in a plastic tube containing 1 ml of 0.5 M EDTA and was stored on ice until plasma preparation, after which they were stored at -80°C. Glucose, lactate and creatine kinase activity were measured with a clinical biochemistry automate (COBAS-MIRA Plus, Roche). Cortisol concentration was measured with the automated analyzer Centaur (Siemens) using a kit designed for human serum and that we validated for pig serum.

The PiF1a line consisted of performance tested pigs (n = 208) of the German commercial cross Pietrain × (German Large White × German Landrace). At slaughter in the FBN experimental slaughterhouse the left adrenal gland was dissected, trimmed of visceral fat and weighed.

### Detection of DNA sequence variation and genotyping

Genomic DNA was isolated from skin or liver samples according to the standard phenol-chlorophorm extraction protocol.

DNA sequence variation was detected either *in silico *(*POMC*, *NR3C1*, *AVP*, *CRHR2*) by alignment of available porcine sequences and confirmed by direct sequencing or *de novo *by comparative sequencing of each two individuals of the breeds Pietrain, German Large White, German Landrace and Wild Boar (*CRHR1*, *CRHBP*, *AVPR1B*, *UCN*). The SNPs *MC2R *c.306T>G and *CRH *g.233C>T were published previously by Jacobs *et al. *[30] and Murani *et al. *[29] respectively.

Genotyping of the SNPs *RYR*1 c.1843C>T and *CRH *g.233C>T was performed by PCR-RFLP and SSCP respectively as described previously (D'Eath *et al. *[4] and Murani *et al. *[29] respectively). The polymorphisms *CRHR1 *c.*866_867insA, *CRHBP *c.51G>A, *MC2R *c.306T>G, *NR3C1 *c.*2122A>G, *AVPR1B *c.1084A>G and *UCN *g.1329T>C were genotyped by PCR-RFLP. Briefly, the polymorphic sites were amplified in 20 μl PCR reactions containing 100 ng genomic DNA, 0.2 mM dNTP, primer as listed in Additional File 1 (Table S1), and 0.5 U SupraTherm Taq-polymerase (Ares Biosciences, Köln, Germany). The temperature profile included initial denaturation at 95°C for 3 min, followed by 40 cycles of denaturation at 95°C for 15 s, annealing at the specific temperature (Additional File 1, Table S1) for 60 s, extension at 72°C for 60 s, and one cycle of final extension at 72°C for 5 min. To detect the polymorphisms 10 μl of the amplified DNA were digested using the appropriate enzyme (Additional File 1, Table S1) overnight according to manufacturer recommendations (Fermentas, St. Leon-Rot, Germany), and the resulting RFLP was analyzed on 2% ethidium bromide stained agarose gel.

The SNP *AVP *c.207A>G was genotyped by SSCP. The PCR was performed as described above, only scaled down to 10 μl (Additional File 1, Table S2). PCR products were separated on a 12% (49:1 AA:Bis) PAA gel containing 10% urea at 450 V for 4 hours at room temperature and subsequently visualized by silver staining.

The SNP *CRHR2 *c.*13T>C was genotyped by pyrosequencing. The PCR was performed as described above, only scaled up to 25 μl, using a step-down temperature profile (Additional File 1, Table S3). The subsequent pyrosequencing reaction was performed as described by Srikanchai *et al. *[61].

The insertion/deletion c.293_298del in *POMC *was analyzed as a length polymorphism on a MegaBACE 750 capillary sequencer using the MegaBACE Fragment Profiler v1.2 software (GE Healthcare, Munich, Germany). The PCR was performed as described above, only scaled down to 10 μl, using a step-down temperature profile (Additional File 1, Table S4).

### Statistical analysis

Population genetic analyses were performed using the PowerMarker V3.25 software [62]. Association between the candidate gene DNA markers and phenotypic variation was analyzed using general linear model (Proc GLM, SAS V9.1, SAS Institute, Cary, NC, USA). Genotypes of the *RYR1 *SNP c.1843C>T were considered as a fixed effect in the model for analysis of every other DNA marker because it has been shown to affect stress responsiveness and aggressiveness in previous and also in the present study (see the Results and Discussion section). For the behaviour traits the model included fixed effects of the marker genotype, *RYR1 *c.1843C>T genotype, slaughter batch, sex and treatment and for the physiological stress parameters also the fixed effect of the dam line. Skin lesion scores and creatine kinase concentrations were log_10_-transformed before analysis. For adrenal weight the model included fixed effects of the marker genotype, *RYR1 *c.1843C>T genotype, farm, sex, and body weight as a covariate. Least square means for marker genotypes were compared by a t-test and the p-values were adjusted by Tukey-Kramer correction. False discovery rate (q-value, [39]) was computed using the JMP Genomics 3 software (SAS Institute, Cary, NC, USA).

### Rydiation hybrid physical Mapping

Physical mapping was performed using the INRA-University of Minnesota porcine Radiation Hybrid (IMpRH) panel. The panel was screened by a standard PCR and the products were resolved on 2% agarose gels. The primers and PCR conditions used are detailed in Additional File 1 (Table S5). Regional assignment was obtained using the multipoint analysis option of the IMpRH mapping tool at the IMpRH server http://www.toulouse.inra.fr.

## Authors' contributions

EM performed the statistic analysis and drafted the manuscript. SP assisted the statistic analysis, participated in sampling and assisted drafting the manuscript and revising it critically for scientific content. RBD, SPT and EK designed the mixing experiment and performed lesion scoring. GE, LT and RK provided input to the design of the mixing experiment, and participated in sampling and phenotyping. AF and PM performed measurements of the physiological stress parameters. KW significantly contributed to the concept, design and coordination of the study and in drafting the manuscript. All authors read and approved the final manuscript.

## Supplementary Material

Additional file 1 **Tables S1 to S6**. Primer sequences, PCR and genotyping assay conditions, results of the IMpRH mapping Click here for file
